# Moral resilience, hospital ethical climate, moral courage, and moral sensitivity among intensive care unit nurses: a structural equation model

**DOI:** 10.3389/fpsyg.2026.1803036

**Published:** 2026-06-19

**Authors:** Yuping Xiang, Shouyi He, Ling Zeng

**Affiliations:** Department of Critical Care Medicine, West China Hospital, Sichuan University/West China School of Nursing, Sichuan University, Chengdu, Sichuan, China

**Keywords:** hospital ethical climate, ICU nurses, moral courage, moral resilience, moral sensitivity

## Abstract

**Objective:**

To investigate the association between intensive care unit nurses' moral resilience and the hospital ethical climate, moral courage, and moral sensitivity, and to determine whether moral courage and moral sensitivity mediate the relationship between ethical climate and moral resilience.

**Methods:**

A descriptive, cross-sectional study. Overall, 363 intensive care unit nurses from eight tertiary hospitals in Southwest China were recruited using convenience sampling. The Moral Resilience Scale, Hospital Ethical Climate Scale, Moral Courage Scale, and Moral Sensitivity Scale were used. Data were analyzed using SPSS 26.0 and AMOS 29.0, and mediation was tested using the bootstrap method.

**Results:**

Intensive care unit nurses demonstrated a moderate moral resilience level (49.71 ± 5.53), which was positively correlated with hospital ethical climate (*r* = 0.38, *p* < 0.01) and moral courage (*r* = 0.44, *p* < 0.01), but not with moral sensitivity. Structural equation modeling showed that hospital ethical climate was closely associated wth moral resilience (effect size = 42.86%) and moral courage acted as chain mediators between hospital ethical climate and moral resilience (effect size = 58.93%). The model demonstrated a good fit (χ^2^/df = 2.39, RMSEA = 0.06).

**Conclusion:**

Intensive care unit nurses in Southwest China demonstrated moderate moral resilience. Both hospital ethical climate and moral courage was linker with nurses' moral resilience. Hospitals should improve their ethical climate and provide moral courage training to enhance intensive care unit nurses' moral psychological resources systematically.

**Impact:**

This study identifies key factors influencing moral resilience among intensive care unit nurses, highlights the central role of moral courage in the relationship between hospital ethical climate and moral resilience, and provides practical guidance for nursing managers in developing effective interventions.

## Introduction

The intensive care unit (ICU) is a highly intensive and complex environment within the healthcare system. Nurses must manage frequent changes in patients' conditions and respond to emergencies while navigating challenges such as end-of-life decision-making, resource allocation, communication with families, and ethical conflicts within the care team (Li et al., 2025; Prompahakul et al., 2021). Without adequate organizational support and psychological resources, these stressors may lead to moral dilemmas and emotional exhaustion, ultimately affecting care quality, patient safety, and team stability (Ahmad et al., 2025). Moral resilience, an important protective factor, has gained increasing attention. It refers to an individual's ability to maintain or restore integrity when confronted with moral setbacks or moral distress (MD) (Rushton, 2016). As a key psychological resource, moral resilience strengthens nurses' emotional state awareness and regulation and helps transform negative emotions into inner balance and professional judgment (Varasteh et al., 2025). Therefore, it can effectively mitigate MD and moral injury (Galanis et al., 2025) and reduce burnout and turnover intention (Yi et al., 2024). Rushton (2023) have highlighted that moral resilience development is grounded in several interrelated psychological traits and practical competencies, including the restoration of moral agency, the cultivation of self-management skills, and the enhancement of moral efficacy and self-regulation.

## Moral courage

Moral courage refers to a nurse's willingness and ability to uphold professional ethics and take appropriate action, even in the face of potential negative consequences (such as stress, anxiety, isolation from colleagues, or threats to employment) (Numminen et al., 2019). Moral courage enables nurses to stay true to their personal values while exposing themselves to potential risks and to prioritize patients' safety and dignity over their own sense of safety. In ICU practice, this is demonstrated through actions such as intervening when colleagues act unethically, advocating for appropriate treatment and patient rights, providing effective care to vulnerable groups (e.g., psychiatric or delirious patients), and communicating critical medical information to families (Kleemola et al., 2020). These actions arise from the nurses' strong sense of professional responsibility and motivate decisive action in the face of ethical challenges, thereby enhancing their sense of work meaning and engagement while indirectly improving the overall quality of care (Creedon and Trace, 2025). Moral courage and resilience, as protective factors promoting nurses' well-being, have been reported to be positively associated, with moral courage contributing up to 45% of the variance in resilience (Abdollahi et al., 2021). However, in clinical practice, when risks are combined with other barriers, such as organizational culture, lack of concern from colleagues who lack moral courage, groupthink, or a tendency to redefine unethical behavior as acceptable, nurses may refrain from demonstrating moral courage. This can undermine their commitment to patient care, leading to MD, reduced moral sensitivity, and even moral indifference (Namadi et al., 2023; Lamiani et al., 2017).

Moral sensitivity refers to the nurses' understanding of the patients' vulnerability and their awareness of the ethical implications of decisions made on the patients' behalf (Lützén et al., 2006). Rest (1982) proposed that moral sensitivity is a prerequisite for moral judgment, moral motivation, and moral character, including conviction, courage, perseverance, and the ability to overcome challenges, and is also a key foundation for moral behavior. For ICU nurses, a high level of moral sensitivity supports sound judgment and appropriate decision-making, strengthens confidence in fulfilling professional duties, and enables them to act in accordance with their beliefs, while enhancing their commitment to work and sense of hope (Kraaijeveld et al., 2021; Escolar-Chua, 2018). In contrast, a lack of moral sensitivity may lead to insufficient ethical awareness, resulting in behaviors that deviate from the fundamental principles of nursing ethics. In the conceptual framework proposed by Lützén et al. (2010), MD, ethical climate, and moral sensitivity are interrelated. Within a supportive ethical climate, team members are better able to understand each other's moral and professional responsibilities, thereby fostering moral agency and positive outcomes of moral sensitivity, while also creating an environment that cultivates moral resilience (Weaver et al., 2008; Lützén and Ewalds-Kvist, 2013). Some studies suggest that moral resilience is characterized by the ability to overcome challenging situations in nursing practice, which is a core aspect of moral sensitivity (Üzar Özçetin and Sarioglu, 2022).

The social cognitive theory proposes that individual cognition mediates the influence of environmental conditions on behavioral choices, while behavioral choices, in turn, shape the environment, creating a continuous reciprocal interaction cycle (Aung and Tewogbola, 2019). Within this framework, the hospital's ethical climate, encompassing workplace conditions, organizational values, and team culture, provides a normative foundation for clinical practice while shaping healthcare professionals' perceptions, judgments, and responses when confronted with moral dilemmas (Ammari and Gantare, 2025). The importance of the hospital's ethical climate in shaping nurses' moral beliefs has been confirmed in multiple studies. When nurses are required to demonstrate moral courage, the behavior of other healthcare professionals is an important influencing factor (Kleemola et al., 2020). A positive ethical climate, grounded in shared values, commitments, and principles, as well as ethical standards and laws, influences nurses' moral sensitivity and their approach to addressing ethical issues (Dalmolin et al., 2022; Tang et al., 2023). In addition, workplace culture can foster a sense of psychological safety, thereby positively contributing to nurses' moral resilience (Spilg et al., 2022).

Based on the above, hospital ethical climate, moral courage, and moral sensitivity all play important roles in shaping nurses' moral resilience. However, few studies have examined how nurses truly understand, consciously reflect on, experience, and demonstrate moral resilience during ethical decision-making and practice. Therefore, in this study, we used structural equation modeling to examine the relationships between ICU nurses' moral resilience, hospital ethical climate, moral courage, and moral sensitivity, and to assess the mediating roles of moral courage and moral sensitivity in the relationship between ethical climate and moral resilience. Our objective was to clarify the developmental pathways of moral resilience and provide evidence to guide systematic support strategies for ICU nurses.

This study is grounded in social cognitive theory (Bandura, 2001) as the overarching framework. The theory emphasizes the dynamic interactions between environment, individuals, and behavior, providing a basis for examining how the hospital's ethical climate influences ICU nurses' moral cognition, specifically moral courage and moral sensitivity, and, in turn, promotes the development of a personal trait, namely moral resilience. In addition, Rest's Four-Component Model (Rest, 1982) was adopted as the analytical framework. This model comprises moral sensitivity, moral judgment, moral motivation, and moral character. Among these, moral sensitivity serves as the prerequisite, while moral courage, as a core component of moral character, acts as the driving force. Based on this framework, the following hypotheses were proposed:

Hypothesis 1. The hospital's ethical climate has a direct effect on moral resilience. Hypothesis 2. Moral sensitivity mediates the relationship between the hospital's ethical climate and moral resilience. Hypothesis 3. Moral courage mediates the relationship between the hospital's ethical climate and moral resilience.

## Methods

### Study design and participants

This study employed a cross-sectional survey design and convenience sampling to recruit ICU nurses from eight tertiary hospitals in Southwest China. The inclusion criteria were as follows: working in the ICU for more than 1 year, holding a nursing qualification certificate, and voluntarily participating after providing informed consent. The exclusion criteria included rotating or intern nurses and those not on duty during the survey period, such as nurses attending external training or on leave. The required sample size was calculated using G^*^Power 3.1. Based on multiple linear regression assumptions, with a medium effect size (f^2^ = 0.15), α = 0.05, power of 0.95, and 28 study variables, and accounting for an anticipated 20% rate of invalid responses, a minimum of 304 participants was required.

### Measures

#### Demographic and work-related information

The information included age, gender, only child, marital status, childbearing, education level, years of experience, position, average number of night shifts per month, monthly income and whether the respondent had received ethics courses.

#### Rushton moral resilience scale

The RMRS was developed by Heinze et al. (2021). This 16-items scale comprises four dimensions: response to moral adversity (4 items), relational integrity (5 items), personal integrity (3 items), and moral efficacy (4 items). It uses a 4-point Likert rating scale, with options ranging from “disagree” to “agree” and scores ranging from 1 to 4, some items are reverse-scored. The total scores range from 16 to 64, with higher score indicating higher level of moral resilience. The Cronbach's α of the original scale was 0.84. The RMRS was translated into Chinese by Yang Qingqing et al. in 2022 (Yang et al., 2022), reporting a Cronbach's α of 0.763. In this study, confirmatory factor analysis (CFA) indicated a good model fit with chi-square/degrees of freedom (χ^2^/df) = 1.63, comparative fit index (CFI) = 0.93, Tucker-Lewis index (TLI) = 0.92, and root mean square error of approximation (RMSEA) = 0.04, with an average variance extracted (AVE) of 0.29 and a composite reliability (CR) of 0.85. The Cronbach's α in this study was 0.77.

#### Nurses' moral courage scale

The NMCS was developed by Numminen et al. (2019). The scale consists of 21 items measuring nurses' self-assessed level of moral courage in four dimensions: compassion and true presence (5 items), moral responsibility (4 items), moral integrity (7 items), and commitment to good care (5 items). Responses are recorded on a 5-point Likert scale, where 1 = “Does not in any way describe me” and 5 = “excellently characterizes me.” Total scores range from 21 to 105, with higher scores indicating higher moral courage, The original scale's Cronbach's α was 0.93 The scale was translated and revised into Chinese by Wang Siyao et al. in 2019 (Wang et al., 2019), reporting a Cronbach's α of 0.905 after translation and Cronbach's α coefficients for each dimension ranging from 0.78 to 0.90. In this study, CFA indicated an acceptable model fit with χ^2^/df = 3.57, CFI = 0.91, TLI = 0.89, and RMSEA = 0.08, with an AVE of 0.49 and a CR of 0.95. The Cronbach's α in this study was 0.95.

#### The moral sensitivity questionnaire-revised version into Chinese

The MSQ-R was used to examine the ethical sensitivity of the nurses. Based on a theoretical framework of moral sensitivity (Lützén et al., 2006), The MSQ-R contains nine items measuring three factors: moral burden (4 items), moral strength (3 items) and moral responsibility (3 items). Items are rated on a 6-point Likert scale where 1 means “totally disagree” and 6 means “totally agree.” Total scores range from 9 to 54, with higher scores indicating greater ethical sensitivity. Scores below 32 indicate low moral sensitivity, scores from 32 to 43 indicate moderate moral sensitivity, and scores above 43 indicate high moral sensitivity. Huang et al. (2016) translated and culturally adapted the MSQ-R into Chinese, modifying the original three-factor model into a two-factor model: moral strength and responsibility (5 items), and moral burden (4 items). The overall Cronbach's α for the MSQ-R was 0.820. In this study, CFA showed that the item “When caring for patients, I am always aware of the balance between the potential to benefit them and the risk of causing harm” loaded on both dimensions, moral strength and responsibility and moral burden, leading to unclear conceptual boundaries and poor model fit. Based on theoretical analysis and content review, this item was found to have an overlapping meaning and was removed while retaining the original factor structure. After deletion, the model demonstrated a good fit with χ^2^/df = 2.57, CFI = 0.98, TLI = 0.96, and RMSEA = 0.07, with an AVE of 0.55 and a CR of 0.91. For the individual dimensions, the AVE and CR were 0.58 and 0.85 for moral strength and responsibility, and 0.54 and 0.78 for moral burden. The overall Cronbach's α in this study was 0.868.

#### Hospital ethical climate survey

The HECS was designed to evaluate how the nurses appraised the ethical climate of their workplace (Olson, 1998). The scale consists of 26 items across five dimensions: relations with peers, patients, managers, the hospital and physicians. A 5-point Likert scale is used, with 1 = “almost never true” and 5 = “almost always true”, with higher scores indicates a more positive ethical climate. The overall Cronbach's α of the scale was 0.91, with a test-retest reliability of 0.786 and a content validity index of 0.89. The Chinese version, translated and revised by Wang Lu (Wang, 2018), had a Cronbach's α of 0.92, with dimension-level Cronbach's α coefficients ranging from 0.60 to 0.84. In this study, CFA indicated a good model fit with χ^2^/df = 3.55, CFI = 0.97, TLI = 0.96, and RMSEA = 0.07, with an AVE of 0.50 and a CR of 0.96. The Cronbach's α in this study was 0.95.

#### Procedure

The researchers obtained permission to use the scales from the original authors via email. Data were collected using the Wenjuanxing online survey platform (https://www.wjx.cn/vm/ejLKBA8.aspx).

(https://www.wjx.cn/vm/ejLKBA8.aspx). This study was a cross-sectional survey, and participation involved no physical or psychological risk. Before completing the questionnaire, participants were informed of the study's purpose and significance. All participant information was kept strictly confidential, and to protect the nurses' privacy, the survey was conducted anonymously. Participants were free to withdraw at any time during the survey. Completed questionnaires could be submitted online, and the data collected were used solely for this study. By completing the online survey, participants provided implied consent. The study was conducted in accordance with the principles of the Declaration of Helsinki and was approved by the Ethics Committee of West China Hospital, Sichuan University (Approval No. 2024-2610). A convenience sampling method was used. Eight tertiary hospitals in Southwest China were selected, and each hospital appointed one coordinator to distribute the survey link through WeChat. Data collection occurred from March to May 2025. Subsequently, two researchers performed quality control. All data were checked by two nurses and entered into a computer. Questionnaires showing obvious patterns, errors, or logical inconsistencies, or completed in less than 100 s, were considered invalid and excluded. The survey platform was configured to allow only one submission per ID, and all items were mandatory.

#### Data analysis

Statistical analyses were performed using SPSS 26.0, RStudio, and AMOS 29.0. Normally distributed continuous variables were reported as the mean ± standard deviation, while categorical variables were presented as frequencies and percentages. Independent-sample *t*-tests and analysis of variance (ANOVA) were used to examine differences in moral resilience across demographic and work-related characteristics among ICU nurses. When statistically significant differences were identified, LSD post-hoc tests were performed for multiple comparisons. To assess common method bias, the Unmeasured Latent Method Construct (ULMC) approach was employed. Pearson correlation analysis and RStudio examined the associations among moral resilience, ethical climate, moral courage, and moral sensitivity. A structural equation model was constructed using AMOS 29.0 to explore the pathways linking hospital ethical climate, moral courage, moral sensitivity, and moral resilience. Model fit was evaluated using multiple indices, including χ^2^/df, CFI, TLI, and RMSEA. The smaller the χ^2^/df value, the better the model fit. A smaller RMSEA indicates a better-fitting model. For GFI, TLI, CFI, and AGFI, values range from 0 to 1, with values closer to 1 indicating a better fit. The bias-corrected bootstrap method with 5,000 resamples was applied to test the significance of mediation effects and calculate 95% confidence intervals (CIs).

A *p*-value of less than 0.05 was considered statistically significant.

## Results

## Participants

Overall, 385 questionnaires were collected, of which 22 were excluded. Ultimately, 363 valid responses were included, resulting in a 94.29% effective response rate. Most participants were female (84.0%), held a bachelor's degree (72.45%), worked in general ICUs (49.86%), and with most possessing 1~10 years of clinical experience (74.11%). The majority reported acquiring ethical knowledge primarily through structured training programs (66.67%). These results are set out in Table 1.

**Table 1:** Demographic characteristics (*n* = 363).

Item	Variables	Frequency	Percentage (%)
Gender	Male	58	15.98
	Female	305	84.02
Age	< 25	16	4.41
	25~35	272	74.93
	36~45	66	18.18
	>46	9	2.48
Education level	Junior College	70	19.28
	Bachelor degree	263	72.45
	Master or above	30	8.27
Marital status	Unmarried	111	30.58
	Married	245	67.49
	Other	7	1.93
Number of children	No	181	49.86
	One child	133	36.64
	≥Two child	49	13.50
Department	General ICU	181	49.86
	Medical ICU	36	9.92
	Surgical ICU	88	24.24
	Other ICUs	58	15.98
Years of ICU experience	1~5	149	41.05
	6~10	120	33.06
	11~15	57	15.70
	>16	37	10.19
Position in the hospital	Clinical nurse	276	76.03
	Nurse team leader	62	17.08
	Clinical nurse specialist	21	5.79
	Nurse manager	4	1.10
Monthly income	< 5,000	39	10.74
	5,000~10,000	241	66.39
	>10,000	83	22.87
Average night shifts per month	0	35	9.64
	1~4	56	15.43
	5~10	219	60.33
	>11	53	14.60
Hospitalization history	Yes	182	50.14
	No	181	49.86
method of learning medical ethics	Systematic learning	242	66.67
	Experiential/practical	64	17.63
	Self-directed learning	57	15.70

## Common method bias test

Common method bias was assessed using the ULMC approach. As shown in Table 2, changes in model fit indices were ΔCFI = 0.01 and ΔRMSEA = 0.02. These results indicate that no significant common method bias was present, and it does not compromise the reliability of the study's conclusions.

**Table 2:** Common method bias assessment: ULMC method.

Model	χ^2^/df	CFI	TLI	RMSEA
Baseline measurement model	2.55	0.96	0.96	0.07
ULMC model (with common method factor)	1.88	0.97	0.96	0.05

CFI, Comparative Fit Index; TLI, Tucker-Lewis Index; RMSEA, Root Mean Square Error of Approximation.

## Moral resilience, moral courage, moral sensitivity, and hospital ethical climate

ICU nurses had mean moral resilience scores of 49.71 ± 5.53. Breaking down the scores by dimension, the response to moral adversity dimension mean scores was 12.85 **±** 2.20, the personal integrity dimension mean scores was 8.51 **±** 1.17, the relational integrity dimension mean scores was 15.10 **±** 2.68, the moral efficacy dimension mean scores was 13.35 **±** 1.81. ICU nurses had mean moral courage, and moral sensitivity scores of 85.83 ± 11.59, and 34.25 ± 5.13, respectively. The hospital's ethical climate mean score was 110.60 ± 11.56 (Table 3).

**Table 3:** The scores of moral resilience, moral courage, moral sensitivity, and hospital ethical climate.

Scale	Mean (SD)	Actual score range
Moral resilience	49.71 (5.53)	37~61
Subscales	
Response to moral adversity	12.85 (2.20)	7~16
Personal integrity	8.51 (1.17)	5~11
Relational integrity	15.10 (2.68)	9~20
Moral efficacy	13.35 (1.81)	7~16
Moral courage	85.83 (11.59)	54~105
Subscales	
Moral integrity	28.48 (4.02)	17~35
Commitment to good care	19.91 (3.15)	11~25
Compassion and true presence	20.87 (2.94)	11~25
Moral responsibility	16.56 (2.40)	8~20
Moral sensitivity	34.25 (5.13)	16~45
Subscales	
Moral strength and moral responsibility	23.56 (3.33)	12~30
Moral burden	10.69 (2.46)	3~18
Hospital ethical climate	110.60 (11.56)	64~130
Subscales	
Relations with peers	17.65 (1.82)	11~20
Relations with physicians	24.33 (3.14)	16~30
Relations with patients	17.18 (1.93)	12~20
Relations with managers	26.03 (2.93)	12~30
Relations with the hospital	25.42 (3.04)	13~30

## Differences in moral resilience across demographic characteristics

ANOVA and *t*-test results demonstrated that overall moral resilience scores among ICU nurses varied significantly according to the number of night shifts (*F* = 6.40, *p* < 0.01). Analysis of subdimensions showed that coping with moral adversity differed by monthly income, number of night shifts, and method of learning medical ethics (*F* = 3.55, *p* = 0.03; *F* = 9.85, *p* < 0.01; *F* = 3.64, *p* = 0.03), while personal integrity varied by number of children and ethics learning approach (*F* = 3.52, *p* = 0.03; *F* = 9.85, *p* = 0.05), and moral efficacy differed by age, number of children, and years of ICU experience (*F* = 9.88, *p* = 0.02; *F* = 8.34, *p* < 0.01; *F* = 4.76, *p* = 0.03) (Supplementary Table S1).

## Correlations between moral resilience, moral courage moral sensitivity, and hospital ethical climate

**Figure 1:**
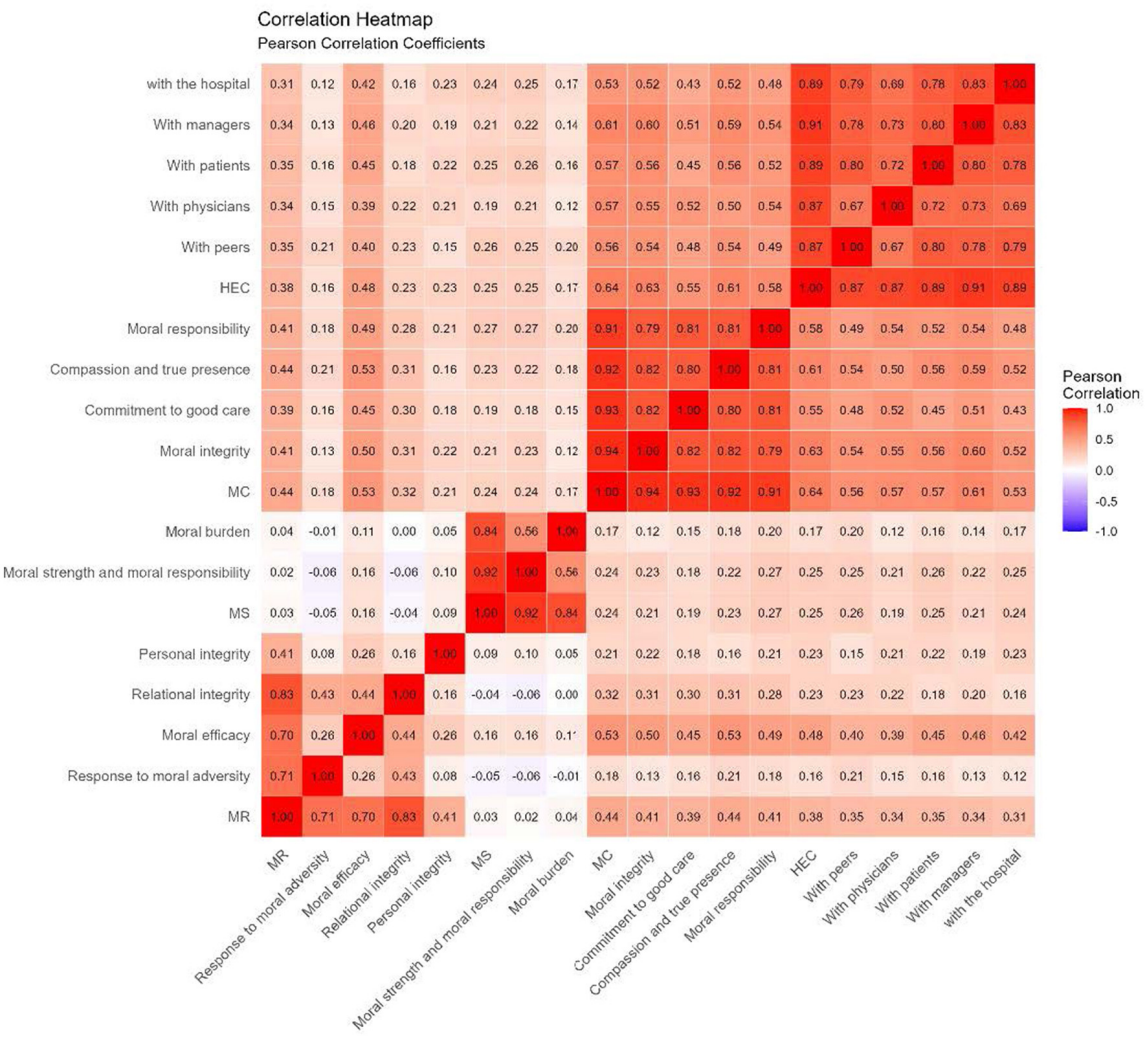
Correlation heatmap.

Pearson correlation analysis demonstrated that ICU nurses' moral resilience scores were positively correlated with moral courage and hospital ethical climate (r_s_ = 0.44, *p* < 0.01; r_s_ = 0.38, *p* < 0.01), but were not significantly associated with moral sensitivity (r_s_ = 0.03, *p* = 0.52) (Table 4). The results of correlation analysis of each dimension are shown in Figure 1.

**Table 4:** Correlations of moral resilience in ICU nurses.

Scale	MR	MC	MS	HEC
**MR**	1			
**MC**	0.44^**^	1		
**MS**	0.03	0.24^**^	1	
**HEC**	0.38^**^	0.64^**^	0.25^**^	1

^**^p < 0.01; MR, Moral Resilience; MC, Moral Courage; MS, Moral Sensitivity; HEC, Hospital Ethical Climate.

## Structural equation modeling results

**Figure 2:**
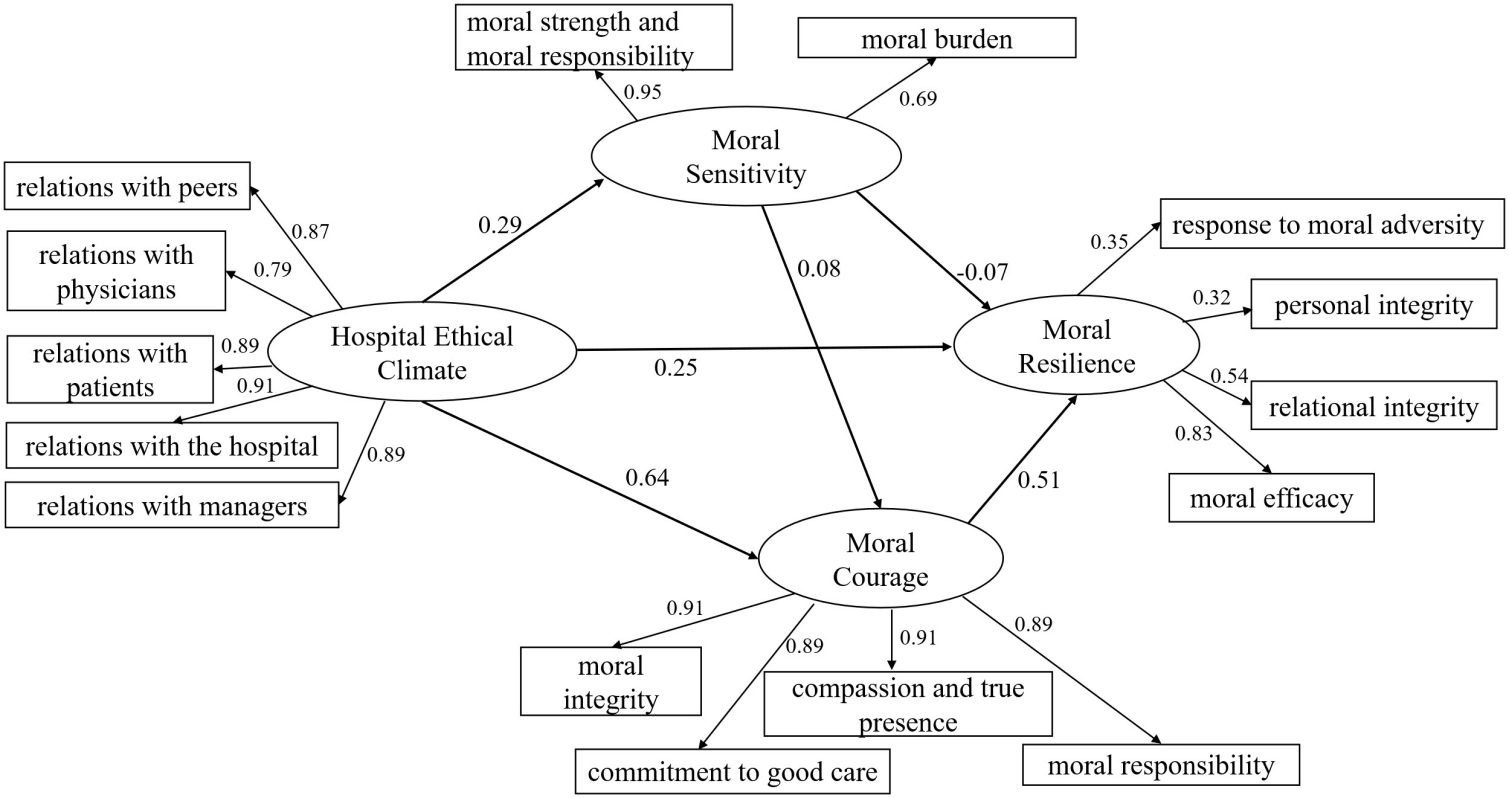
Structural equation model (with covariates).

A structural equation model was constructed with hospital ethical climate as the independent variable, moral courage and moral sensitivity as mediators, and moral resilience as the dependent variable (Figure 2). Maximum likelihood estimation assessed model fit, and the Chi-square or degrees of freedom (x^2^/df = 2.39, NFI = 0.94, IFI = 0.96, CFI = 0.96, RMSEA = 0.06, ratio fell within an acceptable range, indicating a good fit. Path analysis showed that both hospital ethical climate and moral courage were associated with ICU nurses' moral resilience (Table 5).

**Table 5:** Path coefficient analysis.

Path	B	β	SE	*t*	*p*
HEC → MR	0.11	0.24	0.04	2.85	< 0.01^**^
MC → MR	0.19	0.51	0.04	4.81	< 0.01^**^
MS → MR	−0.03	−0.07	0.02	−0.11	0.27

^**^p < 0.01; MR, Moral Resilience; MC, Moral Courage; MS, Moral Sensitivity; HEC, Hospital Ethical Climate.

Given that the results of the analysis of variance indicated that the number of night shifts had a significant effect on both the total score for moral resilience and the dimension of coping with adversity, and that it was the job characteristic variable most likely to produce confounding effects in theory, this study included it as a covariate in the final structural equation model. To further examine the significance of the mediating effects, bias-corrected bootstrap analysis with 5,000 resamples was conducted to generate 95% confidence intervals (CIs). The results demonstrated that the 95% CIs for total, direct, and indirect effects did not include 0, and the direct effect was smaller than the total one, suggesting a partial mediation model. Specifically, moral courage partly mediated the relationship between hospital ethical climate and moral resilience, with a 0.33 (*p* < 0.01) mediating effect, accounting for 58.93 of the total effect (Table 6).

**Table 6:** Mediating effects of the final model (controlled for the number of night shift).

Structural path	β	SE	95%CI	*p*	Effect size (%)
Direct effect					
HEC → MR	0.24	0.08	(0.09, 0.38)	< 0.01^**^	42.86
Indirect effect					
HEC → MC → MR	0.33	0.06	(0.23, 0.46)	< 0.01^**^	58.93
HEC → MS → MR	−0.02	0.02	(−0.07, 0.03)	0.33	
HEC → MS → MC → MR	0.01	0.01	(0.00, 0.04)	0.12	
**Total effect**	0.56	0.05	(0.45, 0.65)	< 0.01^**^	
**Indirect effect**	0.32	0.06	(0.22, 0.44)	< 0.01^**^	57.14

(1) ^**^p < 0.01. (2)MR, Moral Resilience; MC, Moral Courage; MS, Moral Sensitivity; HEC, Hospital Ethical Climate. (3) The sum of direct and indirect effect percentages (42.86% + 58.93% = 101.79%) exceeds 100% due to rounding of the standardized coefficients (β) used in the calculation. The remaining −1.79% is attributed to the non-significant indirect paths involving moral sensitivity (HEC → MS → MR and HEC → MS → MC → MR).

## Covariate control and robustness checks

Since other demographic variables (monthly income, age, number of children, years of ICU experience, and method of learning medical ethics) only influenced specific sub-dimensions and their simultaneous inclusion led to an overly complex model, their robustness was tested using a stepwise inclusion method. The results showed that, compared with the baseline model that does not include covariates, the mediation effects of the core path coefficients remained largely unchanged, indicating that the study's findings are robust and reliable (Table 7).

**Table 7:** Robustness check results: mediation effects with covariates.

Covariates	Structural path	β	SE	95%CI	*p*
No covariates (baseline)	HEC → MR	0.25	0.07	(0.11, 0.39)	< 0.01
	HEC → MC → MR	0.32	0.05	(0.23, 0.44)	
Number of night Shift	HEC → MR	0.24	0.08	(0.09, 0.38)	
	HEC → MC → MR	0.33	0.06	(0.23, 0.46)	
Learning method	HEC → MR	0.24	0.07	(0.10, 0.39)	
	HEC → MC → MR	0.32	0.05	(0.22, 0.44)	
Monthly income	HEC → MR	0.25	0.07	(0.11, 0.39)	
	HEC → MC → MR	0.32	0.05	(0.22, 0.44)	
Age	HEC → MR	0.24	0.07	(0.10, 0.39)	
	HEC → MC → MR	0.31	0.05	(0.22, 0.44)	
Number of children	HEC → MR	0.24	0.07	(0.10, 0.39)	
	HEC → MC → MR	0.30	0.05	(0.20, 0.42)	
Years of ICU experience	HEC → MR	0.25	0.07	(0.10, 0.39)	
	HEC → MC → MR	0.31	0.05	(0.21, 0.43)	

(1) MR, Moral Resilience; MC, Moral Courage; MS, Moral Sensitivity; HEC, Hospital Ethical Climate. (2) Only direct and primary indirect paths are shown for brevity. All other indirect paths (HEC → MS → MR, HEC → MS → MC → MR) remained non-significant across covariates. (3) The first row (“No Covariates”) presents the baseline model results for comparison.

## Discussion

This study examined the status of moral resilience among ICU nurses and the direct and indirect effects of moral sensitivity, moral courage, and hospital ethical climate. Three key findings emerged. First, ICU nurses in Southwest China demonstrated moderate moral resilience. Second, both hospital ethical climate and moral courage were associated with nurses' moral resilience. Third, moral courage served as a mediator between the hospital's ethical climate and moral resilience.

ICU nurses exhibit moderate moral resilience levels. Although slightly above expectations, this finding aligns with previous studies on ICU nurses in certain regions of China and with Mealer's research (Hu et al., 2024; Mealer et al., 2012b), suggesting that ICU nurses may exhibit higher resilience compared to those in general wards. Several factors may explain this. First, ICUs typically employ team-based scheduling, with nurses managing patients in small groups. Strong team cohesion allows experienced nurses to provide guidance and emotional support when facing moral dilemmas or high-intensity stress, helping individuals maintain psychological stability and moral integrity. Second, in the ICU context, nurse resilience is defined as flexibility, coping ability, and adaptability (Mealer et al., 2012a). Additionally, frequent exposure to moral challenges, while potentially stressful, may offer nurses opportunities to gain experience and enhance their moral resilience. Furthermore, the study found that ICU nurses scored lowest on the personal integrity dimension, which reflects an individual's ability to maintain consistency between values and actions in morally challenging situations (Holtz et al., 2018). Possible reasons include the physician-led medical culture, in which nursing values are often subordinate (McAndrew et al., 2018). Consequently, nurses may adopt a passive or compromising stance in ethical decision-making. Another factor is the frequent occurrence of moral dilemmas, such as futile treatments, which expose nurses to prolonged moral stress from patient suffering and family conflicts (Donkers et al., 2021), which may trigger self-doubt and weaken moral judgment and behavioral consistency. Notably, research suggests that implementing resilience bundles, including moral problem-solving protocols, mindfulness interventions, case discussions, structured reporting, and employee assistance programs, promoting interprofessional ethical collaboration, holding regular nursing ethics committee meetings, and encouraging nurses to develop interests outside of work can help restore nurses' psychological resources (Chen et al., 2025; Davis and Batcheller, 2020). Additionally, the frequency of night shifts was found to affect ICU nurses' moral resilience, a finding that differs from those of previous studies (Chen et al., 2024; Tahghighi et al., 2019). Therefore, to control for potential confusion effects, this study included these factors as covariates in the final model. The results indicate that while the number of night shifts has some influence on moral resilience, it does not alter the core mechanism through which the hospital's ethical climate affects moral resilience. This discrepancy may be due to the uneven distribution of night shifts in this sample, which could exaggerate a spurious statistical association. Nevertheless, night shift frequency may not act in isolation, and its relationship with moral resilience could involve complex pathways, including factors such as workload, which warrants further investigation.

When ICU nurses face moral dilemmas, moral courage enables them to uphold ethical judgments and take appropriate action (Pajakoski et al., 2024). Practicing this courage, especially after repeated successful encounters with ethical challenges, can considerably enhance one's moral efficacy sense, reinforce professional identity, and promote self-fulfillment. The accompanying increase in job satisfaction and professional engagement supports safe, high-quality care and provides a psychological and behavioral basis for moral resilience development. Therefore, moral courage systematic cultivation among ICU nurses is of practical importance. Evidence indicates that moral courage is closely related to nurses' work experience, educational background, frequency of exposure to ethical challenges, and prior ethical training (Hauhio et al., 2021). ICU nurses are encouraged to strengthen moral courage through continuing education to improve academic and ethical competence, regular guidance from senior nurses, and focused training in emotional regulation and communication skills (Huang et al., 2025).

A positive hospital ethical climate promotes moral resilience through several mechanisms. Supportive nurse–patient relationships, strong team backing, and systematic organizational safeguards together help reduce nurses' exposure to MD (Fradelos et al., 2021). Clear ethical guidelines, open discussion forums, and decision-making support further increase nurses' confidence in managing ethical dilemmas (Rushton, 2016; Cooper et al., 2022). Importantly, an ethical climate depends on both formal institutional structures and everyday interpersonal interactions and relationship building. Research indicates that nurses' perceptions of the ethical climate are largely shaped by supportive interactions among colleagues (Spilg et al., 2022) and that strong professional trust and a shared moral mission significantly strengthen their confidence in confronting challenges. Accordingly, while top-down strategies such as culture shaping, managerial leadership, and policy implementation are essential for fostering an ethical climate, bottom-up efforts similarly require emphasis. These include team ethical reflection, interdisciplinary dialogue, and the development of trust-based collaborative networks (Spilg et al., 2022; Yu et al., 2025). Together, institutional policies and organizational culture provide sustained safeguards that enable ICU nurses to uphold professional values and maintain moral integrity in clinical practice.

Research shows that the hospital's ethical climate is closely associated with ICU nurses' moral resilience, with moral courage serving as a mediation factor, highlighting how organizational environments shape behavioral outcomes via individual traits. A positive ethical climate provides direct environmental support while simultaneously fostering nurses' moral courage, which increases their willingness and ability to act on ethical principles and becomes a key driver of moral resilience (Yang et al., 2023). Moreover, moral resilience is not a fixed trait but a psychological capacity that can be nurtured within supportive environments (Young and Rushton, 2017). Therefore, efforts to strengthen ICU nurses' moral resilience should focus on continuous improvement of the hospital's ethical climate alongside targeted programs to reinforce ethical competencies. By integrating organizational resources with individual psychological resources, such initiatives can systematically enhance ICU nurses' adaptability and psychological recovery in high-pressure settings.

Further, this study found that moral sensitivity was not correlated with ICU nurses' moral resilience and did not play a mediating role. This finding differs somewhat from the expectations for the Four-Component Model, which suggests that moral sensitivity, as the ability to recognize ethical issues, is a prerequisite for moral judgment, motivation, and character, and should indirectly influence psychological resources such as resilience through moral cognitive processes (Milliken, 2018). However, the results suggest that this pathway may be altered in the high-pressure ICU environment. ICU nurses are frequently exposed to similar high-pressure environments, which may lead to a homogenization of moral sensitivity and reduced individual variation, or to an elevated threshold for perceiving ethical issues, thereby weakening the observed association. Furthermore, moral sensitivity is a multidimensional construct, and different dimensions may exert opposing effects on resilience. The measurement tool used in this study included moral burden, moral responsibility and moral strength, and nurses with different trait profiles may score differently across these dimensions. Moderate moral sensitivity can help nurses recognize and manage risks, indirectly supporting psychological stability. In contrast, excessive sensitivity, particularly when expressed as a high level of moral burden, may exacerbate MD if not effectively regulated, thereby offsetting the potential positive effects of moral responsibility and weakening its theoretical contribution to resilience (Kovanci and Atli Özbaş, 2024; Palazoglu and Koç, 2019). The coexistence and partial offsetting of these positive and negative effects may obscure any overall association between moral sensitivity and moral resilience. Although this study did not confirm the expected theoretical pathway, it highlights the limitations of applying existing theories in specific professional contexts. Future research could examine the dimensional structure of moral sensitivity and use qualitative methods or latent class analysis to explore its heterogeneity among ICU nurses and its complex relationship with moral resilience.

This study examined how the hospital's ethical climate influences ICU nurses' moral resilience and confirmed the key mediating role of moral courage, providing important implications for both clinical nurses and nursing management. From a theoretical perspective, it developed and validated a model linking hospital ethical climate, moral courage, and moral resilience, demonstrating that organizational environments can shape psychological resilience by strengthening individual psychological qualities and clarifying that moral resilience is a psychological capacity that can be cultivated rather than a fixed trait. From a practical perspective, the study offers guidance for managers when formulating psychological support strategies for ICU nurses. Beyond improving the overall ethical climate, healthcare institutions should implement interventions aimed at fostering moral courage, thereby enhancing nurses' ability to uphold ethical standards and maintain resilience in high-pressure settings. These efforts can, in turn, contribute to stable ICU staffing, patient safety, and high-quality care.

## Limitation

This study has several limitations. First, all participants were nurses from Sichuan Province, which may limit the representativeness of the sample and reduce the generalizability of the findings to other regions. Future studies could adopt multicenter designs involving hospitals across multiple regions to enhance the applicability of the results. Second, regarding the measurement instruments, CFA of the moral resilience scale showed good internal consistency among items, with a CR of 0.91, although the AVE was slightly low. This may be partly due to cross-cultural adaptation of the scale and partly attributable to self-report bias, as responses may be influenced by the participants' psychological state during completion, such as occupational burnout or time pressure, as well as social desirability, potentially introducing measurement error. In addition, one item was removed from the moral sensitivity questionnaire. Although this improved the model fit, it may have affected the completeness of the scale. Future research could incorporate qualitative interviews, additional assessment tools, and multi-method data collection to further validate the structural validity of the scales in larger samples. Finally, this study employed a cross-sectional design, which does not allow for the assessment of dynamic changes or causal relationships between variables. Future research could use longitudinal or intervention-based designs to better explore the developmental mechanisms underlying these constructs.

## Conclusion

ICU nurses' moral resilience was generally moderate and positively correlated with both hospital ethical climate and moral courage, but not with moral sensitivity. Path analysis indicated that the hospital's ethical climate was a significant positive predictor of moral resilience while influencing it indirectly through moral courage, suggesting that the organizational environment enhances resilience primarily by strengthening individuals' willingness and ability to act on ethical principles. To effectively promote ICU nurses' moral resilience, healthcare institutions should both optimize the overall ethical climate and implement targeted training programs focused on moral courage. By integrating organizational support with individual psychological resources, such initiatives can provide systematic support for nurses in upholding professional values.

## Data Availability

The original contributions presented in the study are included in the article/Supplementary material, further inquiries can be directed to the corresponding author.

## References

[B1] Abdollahi R. Iranpour S. Ajri-Khameslou M. (2021). Relationship between resilience and professional moral courage among nurses. J. Med. Ethics Hist. Med. 14:3. doi: 10.18502/jmehm.v14i3.5436 34849212 PMC8595071

[B2] Ahmad A. M. Bani-Issa W. Refaat F. Al-Tamimi M. S. Al-Yafeai T. M. (2025). Moral distress and intention to leave among intensive care unit nurses in the United Arab Emirates. Int. J. Nurs. Sci. 12, 581–587. doi: 10.1016/j.ijnss.2025.08.003 41367589 PMC12684761

[B3] Ammari N. Gantare A. (2025). Ethical climate and turnover intention among nurses: a scoping review. Nurs. Ethics. 32, 1434–1457. doi: 10.1177/09697330241296875 39487744

[B4] Aung N. Tewogbola P. (2019). The impact of emotional labor on the health in the workplace: a narrative review of literature from 2013-2018. AIMS Public Health. 6, 268–275. doi: 10.3934/publichealth.2019.3.268 31637276 PMC6779598

[B5] Bandura A. (2001). Social cognitive theory: an agentic perspective. Annu. Rev. Psychol. 52, 1–26. doi: 10.1146/annurev.psych.52.1.1 11148297

[B6] Chen J. Lin N. Ye X. Chen Y. Wang Y. Xu H. (2025). Coping strategies and interventions to alleviate moral distress among pediatric ICU nurses: a scoping review. Nurs. Ethics. 32, 437–459. doi: 10.1177/09697330241252875 38749499

[B7] Chen X. Zhang Y. Zheng R. Hong W. Zhang J. (2024). Latent profiles of nurses' moral resilience and compassion fatigue. Nurs. Ethics. 31, 635–651. doi: 10.1177/09697330231222594 38148631

[B8] Cooper A. L. Leslie G. D. Brown J. A. (2022). Defining the influence of external factors on nurse resilience. Int. J. Ment. Health Nurs. 31, 1523–1533. doi: 10.1111/inm.13059 36008889 PMC9805183

[B9] Creedon S. Trace A. (2025). From protection of sacrificial self to critical turning points and growth: redeployed nurses' experiences on the frontline during the COVID-19 pandemic. PLoS ONE. 20:e0314830. doi: 10.1371/journal.pone.0314830 40839593 PMC12370062

[B10] Dalmolin G. L. Lanes T. C. Bernardi C. M. S. Ramos F. R. S. (2022). Conceptual framework for the ethical climate in health professionals. Nurs. Ethics. 29, 1174–1185. doi: 10.1177/09697330221075741 35545250

[B11] Davis M. Batcheller J. (2020). Managing moral distress in the workplace: creating a resiliency bundle. Nurse Lead. 18, 604–608. doi: 10.1016/j.mnl.2020.06.007 32837357 PMC7391064

[B12] Donkers M. A. Gilissen V. Candel M. van Dijk N. M. Kling H. Heijnen-Panis R. . (2021). Moral distress and ethical climate in intensive care medicine during COVID-19: a nationwide study. BMC Med. Ethics. 22:73. doi: 10.1186/s12910-021-00641-3 34139997 PMC8211309

[B13] Escolar-Chua R. L. (2018). Moral sensitivity, moral distress, and moral courage among baccalaureate Filipino nursing students. Nurs. Ethics. 25, 458–469. doi: 10.1177/0969733016654317 27364536

[B14] Fradelos E. C. Latsou D. Alikari V. Papathanasiou I. V. Roupa A. Balang V. . (2021). Greek nurses' perception of hospital ethical climate: a cross-sectional study. Adv. Exp. Med. Biol. 1337, 17–25. doi: 10.1007/978-3-030-78771-4_3 34972887

[B15] Galanis P. Iliopoulou K. Katsiroumpa A. Moisoglou I. Igoumenidis M. (2025). Moral resilience protects nurses from moral distress and moral injury. Nurs. Ethics. 32, 1617–1628. doi: 10.1177/09697330251324298 40019376

[B16] Hauhio N. Leino-Kilpi H. Katajisto J. Numminen O. (2021). Nurses' self-assessed moral courage and related socio-demographic factors. Nurs. Ethics. 28, 1402–1415. doi: 10.1177/0969733021999763 34100317

[B17] Heinze K. E. Hanson G. Holtz H. Swoboda S. M. Rushton C. H. (2021). Measuring health care interprofessionals' moral resilience: validation of the Rushton Moral Resilience Scale. J. Palliat. Med. 24, 865–872. doi: 10.1089/jpm.2020.0328 33196347

[B18] Holtz H. Heinze K. Rushton C. (2018). Interprofessionals' definitions of moral resilience. J. Clin. Nurs. 27, e488–e494. doi: 10.1111/jocn.13989 28771909

[B19] Hu M. Zhang H. Wu C. Li L. Liang X. Zhang Y. . (2024). Relationship between moral resilience and secondary traumatic stress among ICU nurses: a cross-sectional study. Nurs. Crit. Care. 29, 1363–1372. doi: 10.1111/nicc.13120 39072948

[B20] Huang F. F. Yang Q. Zhang J. Zhang Q. H. Khoshnood K. Zhang J. P. (2016). Cross-cultural validation of the moral sensitivity questionnaire-revised Chinese version. Nurs. Ethics. 23, 784–793. doi: 10.1177/0969733015583183 26002939

[B21] Huang M. Guo J. Li H. Li M. Huang C. Ding S. . (2025). Latent profiles of nursing students' moral courage, moral sensitivity, and moral resilience: a cross-sectional study. BMC Nurs. 24:857. doi: 10.1186/s12912-025-03528-5 40619384 PMC12232737

[B22] Kleemola E. Leino-Kilpi H. Numminen O. (2020). Care situations demanding moral courage: content analysis of nurses' experiences. Nurs. Ethics. 27, 714–725. doi: 10.1177/0969733019897780 31984838

[B23] Kovanci M. S. Atli Özbaş A. (2024). Moral distress and moral sensitivity in clinical nurses. Res. Nurs. Health. 47, 312–323. doi: 10.1002/nur.22366 38142307

[B24] Kraaijeveld M. I. Schilderman J. H. van Leeuwen E. (2021). Moral sensitivity revisited. Nurs. Ethics. 28, 179–189. doi: 10.1177/0969733020930407 32787609

[B25] Lamiani G. Borghi L. Argentero P. (2017). When healthcare professionals cannot do the right thing: a systematic review of moral distress and its correlates. J. Health Psychol. 22, 51–67. doi: 10.1177/1359105315595120 26220460

[B26] Li L. Zou X. Chen H. (2025). Workload in ICU nurses: a systematic review and meta-analysis of the Nursing Activities Score. Intensive Crit. Care Nurs. 91:104086. doi: 10.1016/j.iccn.2025.104086 40483752

[B27] Lützén K. Blom T. Ewalds-Kvist B. Winch S. (2010). Moral stress, moral climate and moral sensitivity among psychiatric professionals. Nurs. Ethics. 17, 213–224. doi: 10.1177/0969733009351951 20185445

[B28] Lützén K. Dahlqvist V. Eriksson S. Norberg A. (2006). Developing the concept of moral sensitivity in health care practice. Nurs. Ethics. 13, 187–196. doi: 10.1191/0969733006ne837oa 16526152

[B29] Lützén K. Ewalds-Kvist B. (2013). Moral distress and its interconnection with moral sensitivity and moral resilience: viewed from the philosophy of Viktor E. Frankl. J. Bioeth. Inq. 10, 317–324. doi: 10.1007/s11673-013-9469-0 23856882

[B30] McAndrew N. S. Leske J. Schroeter K. (2018). Moral distress in critical care nursing: the state of the science. Nurs. Ethics. 25, 552–570. doi: 10.1177/0969733016664975 27660185

[B31] Mealer M. Jones J. Moss M. (2012a). A qualitative study of resilience and posttraumatic stress disorder in United States ICU nurses. Intensive Care Med. 38, 1445–1451. doi: 10.1007/s00134-012-2600-6 22618093

[B32] Mealer M. Jones J. Newman J. McFann K. K. Rothbaum B. Moss M. (2012b). The presence of resilience is associated with a healthier psychological profile in intensive care unit (ICU) nurses: results of a national survey. Int. J. Nurs. Stud. 49, 292–299. doi: 10.1016/j.ijnurstu.2011.09.015 21974793 PMC3276701

[B33] Milliken A. (2018). Nurse ethical sensitivity: an integrative review. Nurs. Ethics. 25, 278–303. doi: 10.1177/0969733016646155 27230913

[B34] Namadi F. Shahbaz A. Jasemi M. (2023). Nurses' lived experiences of moral courage inhibitors: a qualitative descriptive study. SAGE Open Nurs. 9:23779608231157326. doi: 10.1177/23779608231157326 36844423 PMC9944332

[B35] Numminen O. Katajisto J. Leino-Kilpi H. (2019). Development and validation of Nurses' Moral Courage Scale. Nurs. Ethics. 26, 2438–2455. doi: 10.1177/0969733018791325 30185132

[B36] Olson L. L. (1998). Hospital nurses' perceptions of the ethical climate of their work setting. Image J. Nurs. Sch. 30, 345–349. doi: 10.1111/j.1547-5069.1998.tb01331.x 9866295

[B37] Pajakoski E. Leino-Kilpi H. Stolt M. Cartolovni A. Suhonen R. (2024). Nurses' justifications for morally courageous acts in ethical conflicts: a narrative inquiry. Nurs. Ethics. 26:9697330241284357. doi: 10.1177/09697330241284357 39325973 PMC11993820

[B38] Palazoglu C. A. Koç Z. (2019). Ethical sensitivity, burnout, and job satisfaction in emergency nurses. Nurs. Ethics. 26, 809–822. doi: 10.1177/0969733017720846 28814140

[B39] Prompahakul C. Keim-Malpass J. LeBaron V. Yan G. Epstein E. G. (2021). Moral distress among nurses: a mixed-methods study. Nurs. Ethics. 28, 1165–1182. doi: 10.1177/0969733021996028 33888021

[B40] Rest J. R. (1982). A psychologist looks at the teaching of ethics. Hastings Cent. Rep. 12, 29–36. doi: 10.2307/3560621 7068368

[B41] Rushton C. H. (2016). Moral resilience: a capacity for navigating moral distress in critical care. AACN Adv. Crit. Care. 27, 111–119. doi: 10.4037/aacnacc2016275 26909461

[B42] Rushton C. H. (2023). Transforming moral suffering by cultivating moral resilience and ethical practice. Am. J. Crit. Care. 32, 238–248. doi: 10.4037/ajcc2023207 37391375

[B43] Spilg E. G. Rushton C. H. Phillips J. L. Kendzerska T. Saad M. Gifford W. . (2022). The new frontline: exploring the links between moral distress, moral resilience and mental health in healthcare workers during the COVID-19 pandemic. BMC Psychiatry. 22:19. doi: 10.1186/s12888-021-03637-w 34991514 PMC8734541

[B44] Tahghighi M. Brown J. A. Breen L. J. Kane R. Hegney D. Rees C. S. (2019). A comparison of nurse shift workers' and non-shift workers' psychological functioning and resilience. J. Adv. Nurs. 75, 2570–2578. doi: 10.1111/jan.14023 30957259

[B45] Tang F. W. K. Ng M. S. N. Choi K. C. Ling G. C. C. So W. K. W. Chair S. Y. (2023). Impacts of ethical climate and ethical sensitivity on caring efficacy. Nurs. Ethics. 28:9697330231222595. doi: 10.1177/09697330231222595 38155364

[B46] Üzar Özçetin Y. S. Sarioglu G. (2022). The relationship between resilience, moral sensitivity, and cultural competence among nurses. Psychol. Health Med. 27, 1672–1681. doi: 10.1080/13548506.2021.1916955 33870817

[B47] Varasteh S. Nia H. S. Navidhamidi M. Esmaeili M. (2025). Explaining the concept of moral resilience in intensive care unit nurses: a directed content analysis. Nurs. Inq. 32:e12692. doi: 10.1111/nin.12692 39644513

[B48] Wang L. (2018). Chinese translation of the Hospital Ethical Climate Scale and its application research. (master's thesis). Zhengzhou University, Zhengzhou.

[B49] Wang S. Y. Wei L. L. Zhang Y. Liu T. Jiang W. B. Yang H. P. . (2019). Chinese translation of the Nurses' Moral Courage Scale and its reliability and validity testing. J. Nurs. Sci. 34, 92–95. doi: 10.3870.j.issn.1001-4521.2019.21.092

[B50] Weaver K. Morse J. Mitcham C. (2008). Ethical sensitivity in professional practice: concept analysis. J. Adv. Nurs. 62, 607–618. doi: 10.1111/j.1365-2648.2008.04625.x 18355227

[B51] Yang Q. Zheng Z. Ge L. Huang B. X. Liu J. Wang J. . (2023). The impact of resilience on clinical nurses' moral courage during COVID-19: a moderated mediation model of ethical climate and moral distress. Int. Nurs. Rev. 70, 518–526. doi: 10.1111/inr.12871 37584307

[B52] Yang Q. Q. Zheng Z. H. Pang S. Q. Ge L. Huang Y. F. Zhang J. H. . (2022). Chinese translation of the Rushton Moral Resilience Scale and its reliability and validity testing among medical staff. J. Nurs. Sci. 37, 8–11.

[B53] Yi L. Chen Z. Jiménez-Herrera M. F. Gan X. Ren Y. Tian X. (2024). The impact of moral resilience on nurse turnover intentions: the mediating role of job burnout in a cross-sectional study. BMC Nurs. 23:687. doi: 10.1186/s12912-024-02357-2 39334202 PMC11437732

[B54] Young P. D. Rushton C. H. (2017). A concept analysis of moral resilience. Nurs. Outlook. 65, 579–587. doi: 10.1016/j.outlook.2017.03.009 28434608

[B55] Yu Q. Huang C. Yan J. Yue L. Tian Y. Yang J. . (2025). Ethical climate, moral resilience, and ethical competence of head nurses. Nurs. Ethics. 32, 56–70. doi: 10.1177/09697330241230526 38317573

