# Supplementary material for: Moral resilience, hospital ethical climate, moral courage, and moral sensitivity among intensive care unit nurses: a structural equation model
Source: Front Psychol. 2026 Jun 19;17:1803036. doi: 10.3389/fpsyg.2026.1803036 (PMC13327864; doi:10.3389/fpsyg.2026.1803036)
Supplement: Supplementary file 1 [file Table_1.docx]

| **Appendix A Table S1. Differences in Moral Resilience by Demographic Characteristics Among ICU Nurses** | | | | | | | | | | | | |
| --- | --- | --- | --- | --- | --- | --- | --- | --- | --- | --- | --- | --- |
| Variable |  | Moral Resilience | Moral Resilience | | Response to moral adversity | | Personal integrity | | Relational integrity | | Moral efficacy | |
|  |  | M (sd) | t/F | *p* | t/F | *p* | t/F | *p* | t/F | *p* | t/F | *p* |
| Gender | Male | 3.096 (0.353) | -0.256 | 0.798 | 0.845 | 0.399 | 1.398 | 0.163 | -0.520 | 0.603 | -1.954 | 0.051 |
|  | Female | 3.109 (0.345) |  |  |  |  |  |  |  |  |  |  |
| Age | <25 | 2.997 (0.249) | 0.819 | 0.484 | 2.388 | 0.068 | 0.306 | 0.821 | 1.009 | 0.389 | 9.881 | 0.020* |
|  | 25~35 | 3.103 (0.338) |  |  |  |  |  |  |  |  |  |  |
|  | 36~45 | 3.134 (0.385) |  |  |  |  |  |  |  |  |  |  |
|  | >46 | 3.181 (0.423) |  |  |  |  |  |  |  |  |  |  |
| Education level | Secondary technical school | 3.134 (0.314) | 0.746 | 0.475 | 0.560 | 0.571 | 0.684 | 0.505 | 0.548 | 0.579 | 0.141 | 0.868 |
|  | Junior college | 3.093 (0.351) |  |  |  |  |  |  |  |  |  |  |
|  | Bachelor degree | 3.159 (0.366) |  |  |  |  |  |  |  |  |  |  |
| Marital status | Unmarried | 3.051 (0.292) | 2.094 | 0.125 | 0.024 | 0.977 | 2.779 | 0.063 | 2.524 | 0.082 | 2.739 | 0.066 |
|  | Married | 3.132 (0.365) |  |  |  |  |  |  |  |  |  |  |
|  | Other | 3.107 (0.346) |  |  |  |  |  |  |  |  |  |  |
| Childbearing | No | 3.073 (0.312) | 3.871 | 0.144 | 1.276 | 0.280 | 3.516 | 0.031* | 1.165 | 0.313 | 8.342 | <0.001** |
|  | One child | 3.124 (0.362) |  |  |  |  |  |  |  |  |  |  |
|  | >=Two child | 3.185 (0.405) |  |  |  |  |  |  |  |  |  |  |
| Department | General ICU | 3.078 (0.358) | 0.916 | 0.433 | 0.464 | 0.708 | 0.169 | 0.917 | 0.865 | 0.459 | 2.445 | 0.064 |
|  | Medical ICU | 3.141 (0.327) |  |  |  |  |  |  |  |  |  |  |
|  | Surgical ICU | 3.145 (0.325) |  |  |  |  |  |  |  |  |  |  |
|  | Other ICUs | 3.118 (0.346) |  |  |  |  |  |  |  |  |  |  |
| Years of ICU experience | 1~5 | 3.074 (0.331) | 1.754 | 0.156 | 0.220 | 0.883 | 1.520 | 0.209 | 1.852 | 0.137 | 4.760 | 0.003** |
|  | 6~10 | 3.099 (0.325) |  |  |  |  |  |  |  |  |  |  |
|  | 11~15 | 3.144 (0.388) |  |  |  |  |  |  |  |  |  |  |
|  | >16 | 3.208 (0.388) |  |  |  |  |  |  |  |  |  |  |

| **Appendix A Table S1.(continued)** | | | | | | | | | | | | |
| --- | --- | --- | --- | --- | --- | --- | --- | --- | --- | --- | --- | --- |
| Variable |  | Moral Resilience | Moral Resilience | | Response to moral adversity | | Personal integrity | | Relational integrity | | Moral efficacy | |
|  |  | M (sd) | t/F | *p* | t/F | *p* | t/F | *p* | t/F | *p* | t/F | *p* |
| Category of profession | Clinical Nurse | 3.092 (0.319) | 4.053 | 0.256 | 0.591 | 0.621 | 1.037 | 0.376 | 6.230 | 0.101 | 0.834 | 0.476 |
|  | Nurse Team Leader | 3.140 (0.390) |  |  |  |  |  |  |  |  |  |  |
|  | Clinical Nurse Specialist | 3.128 (0.441) |  |  |  |  |  |  |  |  |  |  |
|  | Nurse Manager | 3.453 (0.719) |  |  |  |  |  |  |  |  |  |  |
| Monthly income | <5000 | 3.043 (0.412) | 3.283 | 0.194 | 3.552 | 0.030* | 1.459 | 0.234 | 1.189 | 0.306 | 3.146 | 0.044 |
|  | 5000~10000 | 3.097 (0.328) |  |  |  |  |  |  |  |  |  |  |
|  | >10000 | 3.163 (0.358) |  |  |  |  |  |  |  |  |  |  |
| Average night shifts per month | 0 | 3.186 (0.399) | 6.359 | <0.001** | 9.852 | <0.001** | 2.146 | 0.094 | 2.645 | 0.049 | 1.093 | 0.352 |
|  | 1~4 | 2.977 (0.387) |  |  |  |  |  |  |  |  |  |  |
|  | 5~10 | 3.152 (0.315) |  |  |  |  |  |  |  |  |  |  |
|  | >11 | 3.004 (0.334) |  |  |  |  |  |  |  |  |  |  |
| Hospitalization history | Yes | 3.107 (0.378) | 0.012 | 0.990 | -0.953 | 0.341 | 1.726 | 0.085 | -0.158 | 0.874 | 0.312 | 0.775 |
|  | No | 3.106 (0.311) |  |  |  |  |  |  |  |  |  |  |
| sources of medical ethics knowledge | Systematic Learning | 3.077 (0.346) | 2.759 | 0.065 | 3.636 | 0.027* | 3.105 | 0.046* | 3.041 | 0.049 | 1.849 | 0.159 |
|  | Experiential/Practical | 3.160 (0.352) |  |  |  |  |  |  |  |  |  |  |
|  | Self-directed learning | 3.173 (0.326) |  |  |  |  |  |  |  |  |  |  |
| Note: The results of post-hoc multiple comparisons revealed that:  ① Regarding the total score of moral resilience, significant differences were observed between night shifts of 1–4/month and both 0/month and 5–10/month, as well as between night shifts of >10/month and both **0/month** and 5–10/month.  ② For the response to moral adversity subscale score, significant differences were found between monthly income <¥5,000 and both ¥5,000–¥10,000 and >¥10,000; between night shifts of 1–4/month and both 0/month and 5–10/month; between night shifts of >10/month and both 0/month and 5–10/month; and between ethics knowledge acquired through practice and both systematic learning and self‑directed learning.  ③ For the personal integrity subscale score, significant differences existed between having no children and having one child, as well as between systematic learning of medical ethics knowledge and self‑directed learning.  ④ For the moral efficacy subscale score, significant differences were identified between age >45 years and age 25–35 years; among different childbearing statuses; and between ICU working years >16 years and 1–5 years, 6–10 years, and 11–15 years. | | | | | | | | | | | | |
